# Molecular Characterization of *Eustrongylides* sp. Infecting *Channa punctata* in Bangladesh

**DOI:** 10.1002/vms3.70965

**Published:** 2026-04-29

**Authors:** Salman Shahriar Nibir, Anita Rani Dey, Tanvir Rahman, Julfat Tasnim Suchona, Siyam Hossain, Savayan Sadad Bushra, Jubaida Parveen Juthy, Sandhya Paul, Jarin Tasnim Tanwi, Tamanna Tabassum, Md. Ali Reza Faruk

**Affiliations:** ^1^ Department of Aquaculture Bangladesh Agricultural University Mymensingh Bangladesh; ^2^ Department of Parasitology Bangladesh Agricultural University Mymensingh Bangladesh

**Keywords:** *Channa punctata*, *Eustrongylides* sp, nucleotide polymorphism, phylogeny

## Abstract

**Background:**

*Eustrongylides* (nematode: Dioctophymatidae) are parasitic nematodes of freshwater fishes and fish‐eating birds. It has zoonotic potential and poses a threat to human health, as infection can occur through the consumption of raw or undercooked fish.

**Objectives:**

The present study was designed to characterize *Eustrongylides* sp. isolated from *Channa punctata* using the *Internal Transcribed Spacer 2* (*ITS2*) regions.

**Methods:**

In this study, a total of 137 medium‐sized *C. punctata* (17.5 ± 2.5 cm) were collected from different locations of Jamalpur and Netrokona district, Bangladesh and isolated 44 nematodes. DNA was extracted from the parasites, amplified in the *ITS2* region and sequenced.

**Results:**

All the nematodes were microscopically identified as belonging to the genus *Eustrongylides* based on morphological characteristics, including the presence of inner and outer circle labial papillae at the anterior end and the esophago‐intestinal junction. Initially, the sequences were confirmed up to the lowest taxonomic level possible using Basic Local Alignment Search Tool (BLAST). For phylogenetic analysis, reference sequences of *Eustrongylides* sp. were aligned, and two single nucleotide polymorphisms (SNPs) were detected. The average sequence identity percentage (%) within the studied sequences ranged from 99.7% to 99.8%. A maximum‐likelihood (ML) tree was constructed which showed that the studied sequences clustered within the *Eustrongylides* sp. lineage and grouped with reference sequences, supported by moderate bootstrap values (82%).

**Conclusions:**

Close genetic similarity was observed when compared with reference sequences of *Eustrongylides* sp. Further studies on the investigation of genetic pattern of *Eustrongylides* sp. throughout the country are essential to control this parasite.

## Graphical abstract

In this study, a total of 137 medium‐sized *Channa punctata* (17.5 ± 2.5 cm) were collected from different locations of Jamalpur and Netrokona district, Bangladesh and microscopically identified as belonging to the genus *Eustrongylides* based on morphological characteristics. Two single nucleotide polymorphisms (SNPs) were detected in the studied sequences of *Eustrongylides* sp., and the sequence identity percentage ranged from 99.7% to 99.8%. The phylogenetic analysis showed that the studied sequences clustered within the *Eustrongylides* sp. lineage signifying close genetic relationship.

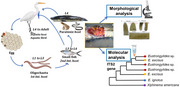

## 1 Introduction

Aquaculture of snakeheads has recently gained significant attention in developing countries such as Thailand, Malaysia, Indonesia, Bangladesh, India and Vietnam for its great economic value (Bich et al. 2020). The unique traits of snakeheads, such as their exceptional air‐breathing capacity and their ability to thrive at high stocking densities, make them a good candidate for aquaculture (Li et al. 2017; Siddaiah et al. 2023). *Channa punctata* (Bloch, 1793) commonly known as spotted snakehead and locally known as ‘Taki’ belonging to family Channidae is well‐known for its taste, high protein content, low intramuscular pin bones and high nutritive value (Bogard et al. 2015; Datta et al. 2013). As a prolific breeder, it is widely cultured and contributes significantly to the aquaculture industry (Kundu and Mandal 2022). Although commercial farming of *C. punctata* has not yet begun in Bangladesh, its strong tolerance to low salinity and stable growth performance show that it holds clear promise for aquaculture development (Dubey et al. 2016). Its palatable taste and processed food products derived from this fish have made it one of the most popular and widely accepted fish species in Bangladesh (Raihan et al. 2020). Being a carnivorous fish (Borman et al. 2015), it prefers to feed on molluscs and insects (Hasan et al. 2025). Due to its mud‐dwelling nature and feeding habits, it can serve as an intermediate host for various nematode parasites, which may lead to significant economic losses in *C. punctata* aquaculture (Kundu and Mandal 2022). Recent studies have also reported several helminth parasites infecting *C. punctata* and emphasized the importance of combined morphological and molecular approaches for their identification (Bari et al. 2024). Consequently, research on fish–parasite interactions is important due to their impacts on fish health and the economic sustainability of aquaculture and fisheries (Dezfuli et al. 2015).

*Eustrongylides* spp., parasitic nematodes from the Dioctophymatidae family, infect various freshwater fish and piscivorous birds (Pekmezci and Bolukbas 2021). They have a heteroxenous life cycle, with aquatic annelids (oligochaetes) serving as the first intermediate host and fish as the second intermediate host (Anderson 2000). *C. punctata* can serve as a paratenic host containing fourth‐stage larvae (L4) of this parasite which reside in abdominal cavity, musculature and ovaries of the fish (Gupta 2019). The larvae are often in encapsulated form or found free in the body cavity of the host (Bjeli‐Cabrilo et al. 2013). The L3 stage of this parasite penetrates the gastrointestinal tract, perforating the intestinal wall to enter the extra‐intestinal space and subsequently migrates to muscular tissue, hepato‐pancreas and gonads of their hosts (Honcharov et al. 2022). Additionally, *Eustrongylides* spp. can pose a zoonotic risk, as consuming raw or undercooked fish containing these larvae can lead to intestinal perforation and gastritis (Eberhard and Ruiz‐Tiben 2014; Ljubojevic et al. 2015). Humans can become accidentally infected by ingesting raw or undercooked freshwater fish that are infected as second intermediate or paratenic hosts. Although human infections are rare, they have been sporadically reported in the United States, South Sudan and various regions of Italy (Castiglione et al. 2023)

Molecular analyses, particularly sequencing of the *Internal Transcribed Spacer* (*ITS*) rDNA region, have proven effective for accurate identification of parasites (Abe 2011; Xiong et al. 2009; Guardone et al. 2021). Mazzone et al. (2019) utilized *ITS‐*based molecular techniques along with anatomical and morphological descriptions to identify both adults and L4 of *Eustrongylides excisus*, whereas Pekmezci and Bolukbas (2021) confirmed that the L4 and adult specimens of *E. excisus* belong to the same taxon through binary genetic distance analysis based on *ITS* regions.

Several studies, including Williams et al. (2022) and Raihan et al. (2020), have reported the presence of *Eustrongylides* larvae in edible fishes using morphological and molecular methods. However, these studies mainly focused on species identification and did not include phylogenetic analysis based on specific geographic areas, such as the two districts examined in the present study. In addition, some studies did not clearly mention the exact locations of the infected fish in Bangladesh. In the present study, the morphological features of the larvae were reassessed before proceeding with molecular analysis. Understanding the phylogenetic relationships among genus *Eustrongylides* is crucial for tracking their evolutionary lineage as well as their potential of disease transmission. Therefore, this study expands upon previous work by not only confirming species identity but also conducting a brief phylogenetic analysis.

## 2 Materials and Methods

### 2.1 Sample Collection

**FIGURE 1:**
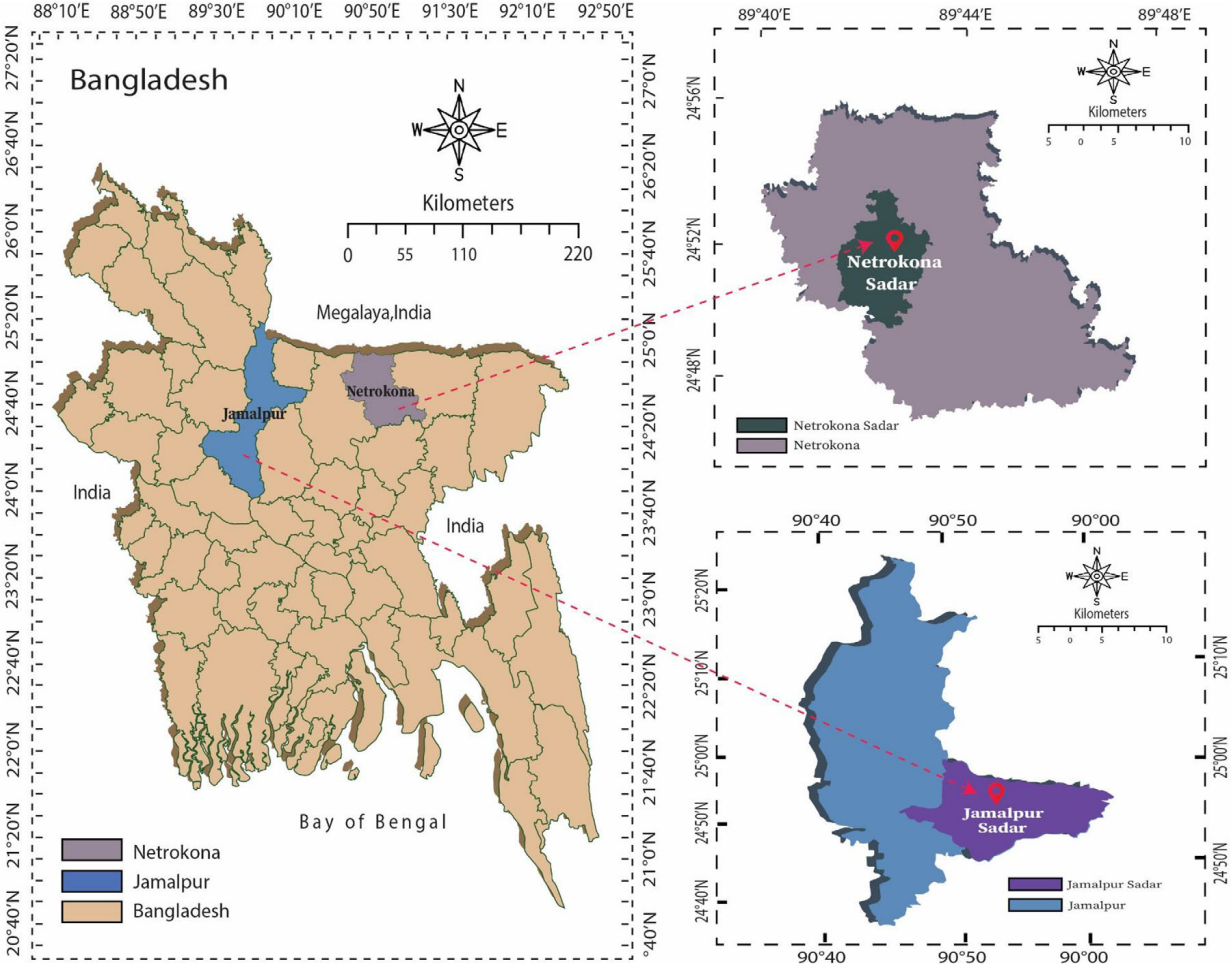
Map of the study area from where the experimental fish were collected.

A total of 137 *C. punctata* specimens were collected from the Jamalpur and Netrokona districts in the Mymensingh division. In Jamalpur, fish were obtained from ‘Farmers Market’ (24.9371° N, 89.9394° E), whereas in Netrokona, specimens were sourced from ‘Mechua Bazar’ (24.8801° N, 90.7299° E) (Figure 1). The selected fish markets are located near rivers, with the Brahmaputra River in Jamalpur and the Magra River in Netrokona. Local fishermen capture various fish, including *C. punctata*, from the natural water bodies such as ditches, beels and stagnant waters formed by these rivers and bring them regularly for sale at local markets. The collected specimens were transported to Bangladesh Agricultural University (BAU) in live condition with sufficient oxygen within the sealed bags, which were placed inside a plastic bucket. The polythene bags were securely tied to prevent fish from escaping with sufficient oxygen inside. Euthanasia of the fish specimens was performed by pithing (quickly) according to UFR‐C Guidelines before dissection, as documented by Yanong et al. (2007). Dissection and isolation of parasites, followed by microscopic examination, were conducted in the Fish Disease Laboratory, Department of Aquaculture and Parasitology Laboratory, Department of Parasitology at BAU.

### 2.2 Isolation of Parasites

For isolation of nematodes, an incision was made along the mid‐ventral line and examining the musculature, visceral organs, mesenteries and body cavities using forceps. Parasites were visible with naked eye within the body cavity, with their heads embedded in the intestines of fish. After isolation, parasites were washed in normal saline for three times.

### 2.3 Microscopic Identification

For microscopic examination, the parasites were placed in Petri dish with lukewarm glycerine alcohol for straightening. Then, temporary slides were prepared using lactophenol, with a cover slip placed over the parasites (Noor et al. 2025). These slides were then examined under a microscope at 10× or 40× magnification. Nematodes were identified according to their key morphological features as described by Kuraiem et al. (2020). The length of hosts (fish) and parasites was measured with measuring scale, and the width of parasites was measured under a microscope with an ocular micrometre. The examined specimens were preserved in absolute alcohol and are deposited in the reference collection of the Fish Disease Laboratory, BAU.

### 2.4 Molecular Identification

#### 2.4.1 Extraction of DNA

A total of 44 *Eustrongylides* sp. specimens were isolated after morphological identification and subjected for DNA extraction. Total genomic DNA was extracted from individual parasite using a commercial kit, QIAamp DNA Mini Kit (Qiagen, Germany), according to the manufacturer's protocol. After measuring the extracted DNA with a nanodrop spectrophotometer, DNA was stored at −20°C until further use.

#### 2.4.2 Amplification of *ITS2* Region and Gel Electrophoresis

A fragment of about ∼900 bp of the *ITS2* region was amplified using the appropriate primers, 18SF (5‐TTGGATGATTCGGTGAGGT‐3) and 28SR (5AACCGCTTAGTAATATGCT‐3) (Xiong et al. 2013). The primers target the *ITS2* region of ribosomal DNA and were selected on the basis of previously published studies and available *Eustrongylides* sequences in GenBank to ensure specific amplification of the target region. The PCR was conducted in a 25 µL reaction volume with 12.5 µL of master mix (containing 0.05 U/µL Taq DNA polymerase, reaction buffer, 0.4 mM of each dNTP and 4 mM MgCl_2_), 2 µL of template DNA (≥30 ng/µL), 1 µL of each primer (10 pmol/µL) and 8.5 µL of double‐distilled water. The amplification profile consisted of an initial denaturation at 95°C for 5 min, followed by 35 cycles of 95°C for 30 s, 55°C for 30 s and 72°C for 30 s, with a final extension at 72°C for 10 min. PCR products were analysed by 2.0% (w/v) agarose gel electrophoresis. A negative control (PCR mixture without template DNA) was included in each amplification run to monitor potential contamination or nonspecific amplification. The amplified positive PCR products of both genes were visualized on 2.0% agarose gel after being stained with Midori Green Advance.

#### 2.4.3 Sequencing

Forty‐four positive PCR products of each isolate were selected before subjecting for sequencing. The PCR products were column purified (Wijard PCR‐Prep, Pro‐mega) before subjecting for sequencing (BigDye Terminator v.3.1 cycle sequencing kit, Applied Biosystem) in an automated sequence (PRISM3730, ABI) using appropriate forward and reverse primers (in separate reactions). Raw sequences were manually inspected and trimmed to remove low‐quality regions at both ends. The edited sequences were aligned using ClustalW implemented in MEGA. After removal of ambiguous sites and gaps, the final alignment length used for phylogenetic analysis was 898 bp. The sequences were compared for nucleotide with those retrieved from GenBank using the Basic Local Alignment Search Tool (BLAST). Species identification was determined from the best‐scoring reference sequence of the BLAST output.

#### 2.4.4 Genetic Data Analysis

The sequences were aligned using the program Clustal W within MEGA software Version 10.2.6 (Kumar et al. 2018). Pairwise comparison was conducted using previously published sequences, and the identity percentages were calculated with them. A maximum‐likelihood (ML) tree was built with 48 sequences, including the studied sequences and 45 reference sequences retrieved from the GenBank database. The phylogenetic reconstruction was performed using the Tamura–Nei nucleotide substitution model. The ML analysis was conducted using the nearest‐neighbour interchange (NNI) heuristic method, and the robustness of the inferred topology was evaluated using 1000 bootstrap replications. *Xiphinema americanum* (KF748494) was selected as the outgroup because it represents a genetically distinct nematode lineage outside the family Dioctophymatidae, allowing appropriate rooting of the phylogenetic tree. Similarly, Zhang et al. (2021) used *X. americanum* to root the phylogenetic trees of *Eustrongylides* sp. The details of reference sequences included in the phylogenetic analysis, specifying the parasite names, host species, references and geographical locations, are provided in Table S1. A total of 44 sequences were submitted in the GenBank database (LC814532‐LC814575).

## 3 Results

### 3.1 Morphological Identification of *Eustrongylides* spp

**FIGURE 2:**
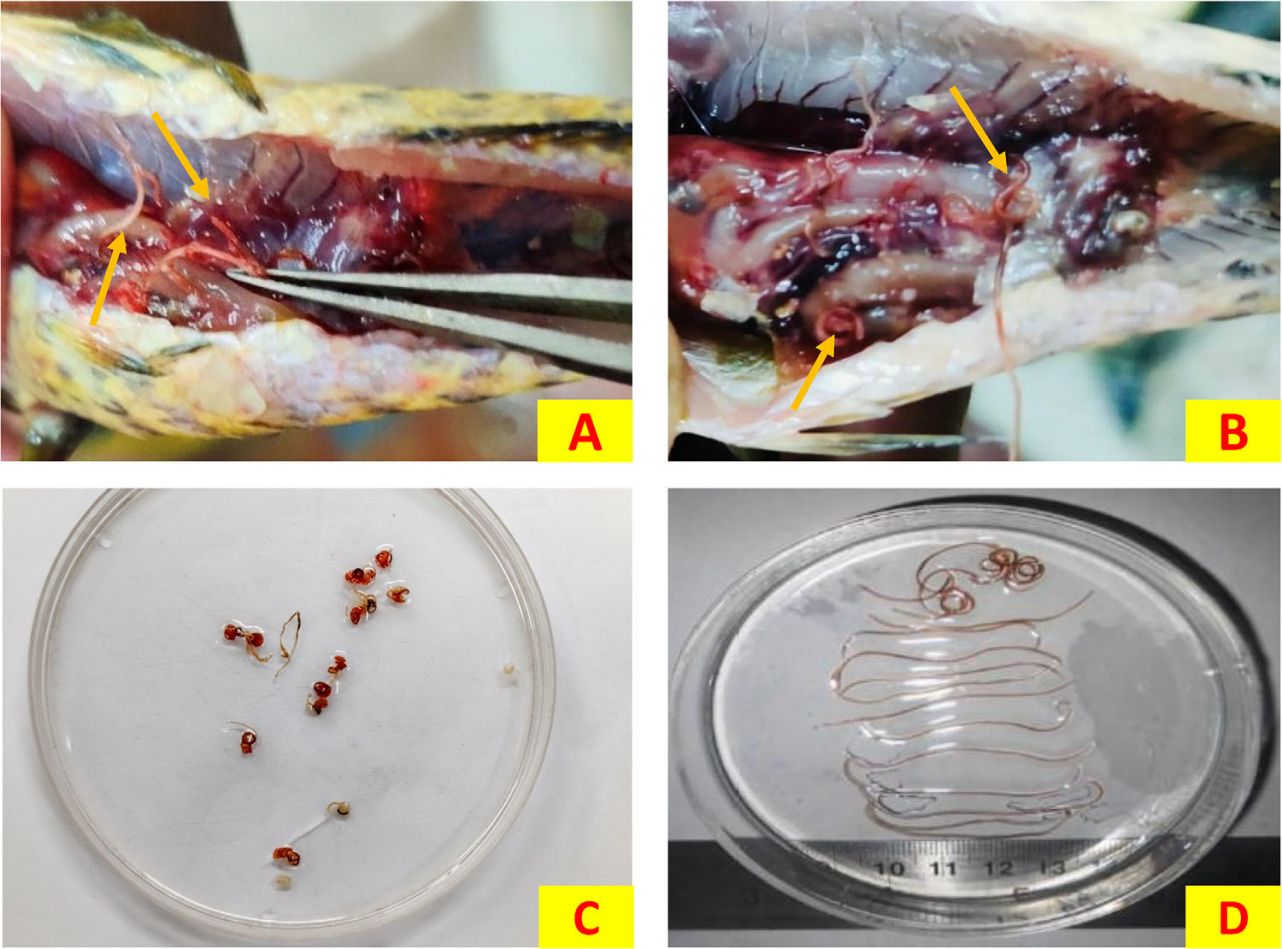
Gross localization and recovery of *Eustrongylides* larvae from *Channa punctata*. (A) Multiple larvae (arrows) observed within the visceral organs during dissection. (B) Coiled larvae (arrows) embedded within the muscle tissue. (C) Recovered larvae following isolation, showing characteristic reddish colouration. (D) Recovered *Eustrongylides* larvae arranged in a Petri dish for gross morphology.

**FIGURE 3:**
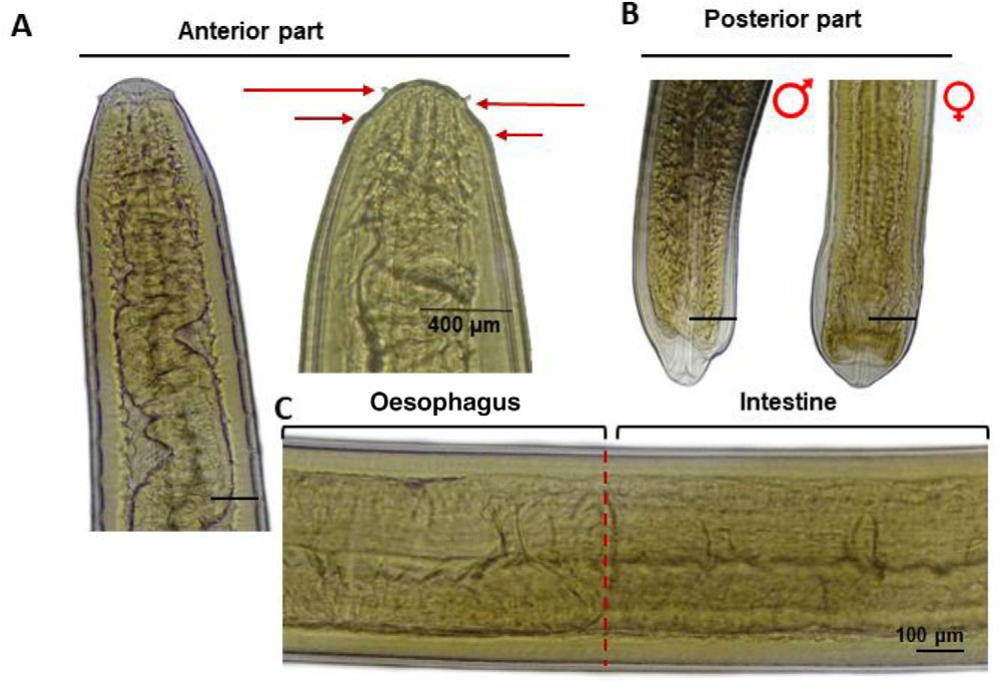
Microscopic features of *Eustrongylides* spp. (A) Two rows of cephalic papillae (short arrow indicates outer and long arrow indicates inner circle papillae) in anterior part (A), posterior part of female and male (B) and oesophago‐intestinal junction (C) at the middle part of the body.

In the present study, 137 medium‐sized (17.5 ± 2.5 cm) *C. punctata* with a mean weight of approximately 66.14 ± 10.02 g were collected from different locations of Jamalpur and Netrokona district, Bangladesh. A total of 44 nematodes were considered for morphological identification as well as molecular characterization. The collected nematodes were observed coiled around the visceral organs, freely within the body cavity, and in some cases embedded within the musculature of the host fish. Most specimens exhibited a characteristic reddish colouration (Figure 2). The parasites had an average length of 48.2 ± 7.5 mm (range: 33.2–63.1 mm) and an average maximum width of 0.6 ± 0.1 mm (range: 0.4–0.8 mm). Microscopic examination showed that the cephalic region had a small oral cavity with two circles of cephalic papillae at the anterior end, arranged as six papillae in the inner circle and six in the outer circle. The papillae in the inner circle had spine‐like sharp apices with narrow bases, whereas the papillae in the outer circle had blunt apices and broader bases. The inner papillae appeared more pointed than those in the outer circle. A long and distinct oesophagus–intestine junction was visible in the mid‐region. The oesophagus measured about 14.6 ± 0.5 mm in length, with a range of 13.25–15.92 mm. The worms had a cuticle with clear transverse striations under the microscope. Male and female L4 of *Eustrongylides* sp. were differentiated according to their morphological differences at the caudal extremity. The male parasites’ posterior ends featured a row of cuticular extensions on the caudal sucker, and its rift was marginally deeper than the female's. On the other hand, posterior end of female parasite was blunt in shape (Figure 3). A comparative summary of the key morphometric and diagnostic features of the recovered L4 larvae alongside previously published descriptions is presented in Table 1. The isolated parasites were tentatively confirmed as fourth‐stage larvae (L4) belonging to the genus *Eustrongylides* Jagerskiold, 1909 by the above‐mentioned morphological features.

**TABLE 1:** Comparative morphometric and diagnostic features of L4 larvae of *Eustrongylides* spp.

Morphological features	Present study	Previous study	Reference
Body length (mm)	48.2 ± 7.5	50–70 (host: *Esox lucius*)	Rahmati‐Holasoo et al. (2024)
Body width (mm)	0.6 ± 0.1	0.395 ± 0.019	Zhang et al. (2021)
Oesophagus length (mm)	14.6 ± 0.5	10–15 (host: *Galaxias maculatus*)	Guagliardo et al. (2019)
Cephalic structures	Small oral opening; cephalic papillae arranged in two concentric circles; inner papillae pointed, outer papillae blunt	Two papillae rows; inner bulged, outer sharp (host: *Danio rerio*)	Fusco et al. (2023)
Male posterior end	Cuticular extensions on caudal sucker	Ventral cleft with cuticular projections on caudal sucker (host: *Sander lucioperca*)	Pekmezci and Bolukbas (2021)
Female posterior end	Blunt	Conical (host: *Hypomesus transpacificus nipponensis*)	Abe (2011)

### 3.2 Molecular Characterization

#### 3.2.1 Species Identification and Genetic Similarity Analysis

After amplifying the *ITS2* region, all field isolates were confirmed as *Eustrongylides* sp. by a distinct ∼900 bp band. Positive PCR products were sequenced, edited and aligned to produce 898 bp. Out of 44 sequences (23 from Jamalpur and 21 from Netrokona), a total of three haplotypes (one from Jamalpur and three from Netrokona) were detected. Overall haplotype and nucleotide diversity of the isolates were 0.0899 and 0.0001, respectively (Table 2). The sequence identities ranged from 99.7% to 99.8% when compared among the studied sequences, 99.8% to 100% when compared with studied sequences and two sequences of *Eustrongylides* sp. (accession nos. GQ215503.1 and GQ215533.1), 97.1% to 97.2% when compared with *E. excisus* (accession nos. MK007967.1 and OP480438.1) and 97.2% to 97.3% when compared with *Eustrongylides ignotus* (accession no. MK340917.1) (Table 3).

**TABLE 2:** Summary of genetic diversity of *Eustrongylides* isolates collected from Jamalpur and Netrokona districts of Bangladesh.

Location	No. of sequence	No. of haplotypes	Haplotype diversity	Nucleotide diversity
Jamalpur	21	03	0.01857	0.00021
Netrokona	23	01	0	0
**Overall**	**44**	**03**	**0.0899**	**0.0001**

*Note*: Haplotype refers to the distinct genetic composition of the studied sequences based on sequence variations, which can be used to differentiate closely related taxa within the *Eustrongylides* genus.

**TABLE 3:** Pairwise nucleotide sequence identity (%) among the studied *Eustrongylides* isolates and selected reference sequences of *Eustrongylides* sp., *Eustrongylides excisus* and *Eustrongylides ignotus* retrieved from GenBank.

S no.	Sequence ID	1	2	3	4	5	6	7	8
1.	LC814539 (studied)	**—**							
2.	LC814550 (studied)	99.7	**—**						
3.	LC814548 (studied)	99.8	99.8	**—**					
4.	GQ215503.1 *Eustrongylides* sp.	99.8	99.8	100	**—**				
5.	GQ215533.1 *Eustrongylides* sp.	99.8	99.8	100	100	**—**			
6.	MK007967.1 *E. excisus*	97.1	97.1	97.2	97.2	97.2	**—**		
7.	OP480438.1 *E. excisus*	97.1	97.1	97.2	97.2	97.2	99.7	**—**	
8.	MK340917.1 *E. ignotus*	97.2	97.2	97.3	97.3	97.3	99.8	99.8	**—**

#### 3.2.2 Nucleotide Distribution

In this study, three haplotypes were aligned with reference sequence of *Eustrongylides* sp. (Accession no.: GQ215503.1) revealing two single nucleotide polymorphisms (SNPs): a transition at position 815 (A ↔ G) and a transversion at position 71 (G ↔ T). The remaining 42 isolates showed no variation at any nucleotide position (Table 4).

**TABLE 4:** Nucleotide variations at specific positions among three haplotypes of *Eustrongylides* isolates from *Channa punctata*, compared with the reference sequence (GQ215503.1).

Haplotype	Nucleotide position		No. of parasites
	73	817	
**GQ215503.1**	G	A	
LC814539	T	.	1
LC814550	.	G	1
LC814548	.	.	42
**Overall**			**44**

*Note*: “.” indicates no nucleotide change from the reference sequence.

#### 3.2.3 Phylogenetic Analysis

The ML tree illustrated the evolutionary relationships among *Eustrongylides* species, with *X. americanum* (KF748494.1) used as the outgroup. The studied sequences (LC814539, LC814548 and LC814550) clustered within the *Eustrongylides* sp. group. Among them, LC814548 formed a sub‐clade with Chinese isolates (e.g., GQ215552.1), supported by a bootstrap value of 82%, whereas LC814550 grouped with sequences from India (KJ458967.1) and China with bootstrap values ranging from 77% to 82%. LC814539 was positioned within the same lineage and supported by a bootstrap value of 82% at the corresponding node.

**FIGURE 4:**
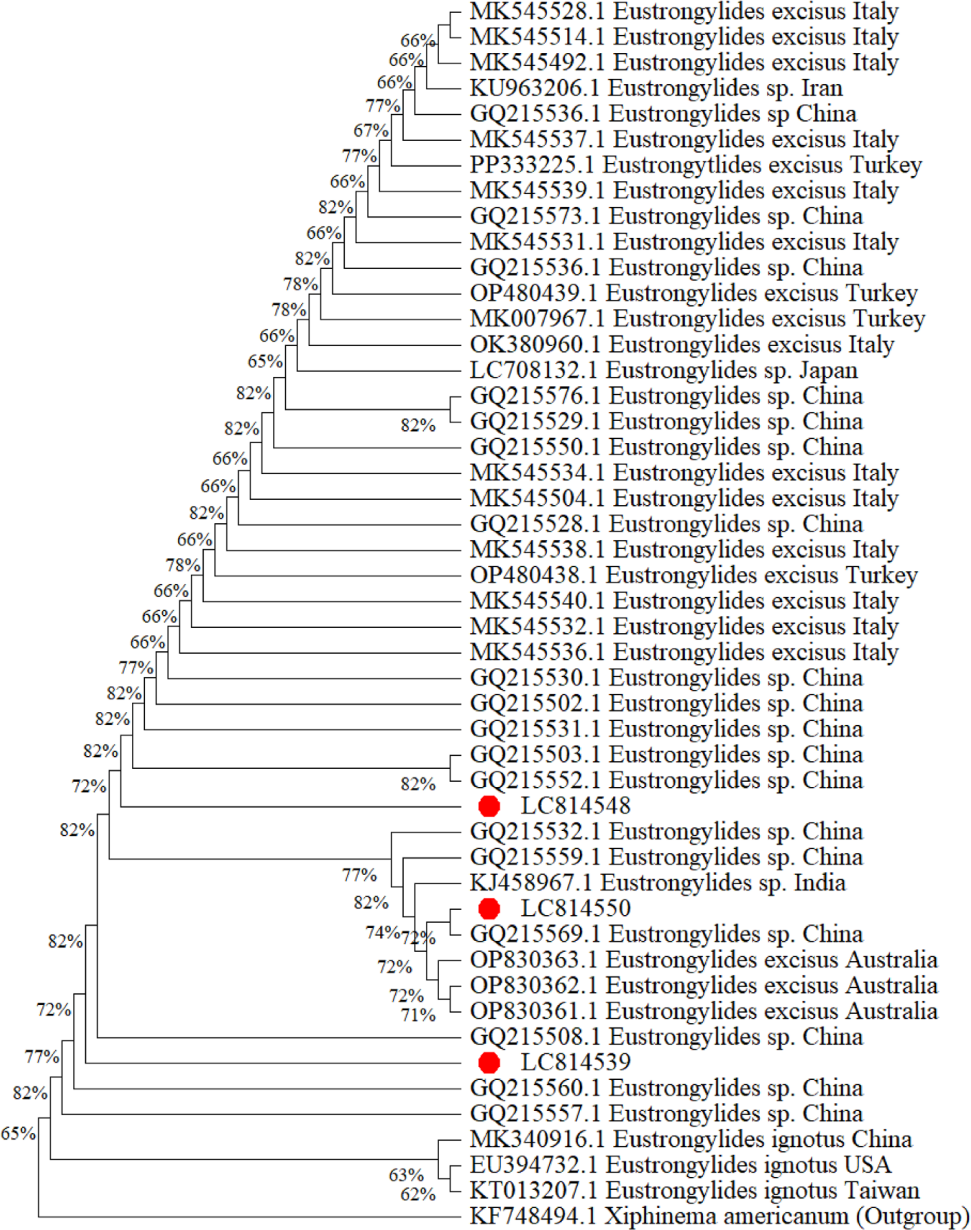
Phylogenetic tree constructed using the maximum‐likelihood (ML) method based on the Tamura–Nei model in MEGA version 12, using 48 sequences. The studied sequences are indicated by red circles.

All studied sequences were clearly separated from the *E. ignotus* lineage, which formed a distinct clade in the analysis. Several *E. excisus* isolates also formed separate clusters, including samples from Italy, Turkey, Japan and Australia, reflecting phylogenetic structuring among major species groups (Figure 4). Although several internal nodes showed moderate bootstrap support, the overall clustering pattern consistently placed the studied isolates within the *Eustrongylides* sp. lineage (Figure 4), supporting their molecular identification at the genus level.

## 4 Discussion

*C. punctata* is an ecologically significant predatory fish, primarily consuming crustaceans, insects, molluscs, fish and organic matter, with minimal plant intake (Hasan et al. 2025). Due to its feeding habits, it is classified as a secondary consumer in the food chain, playing a crucial role in regulating prey populations and influencing aquatic community structure (Bronmark et al. 1992). This species also serves as a paratenic host for *Eustrongylides* spp., a nematode transmitted through trophic interactions (Measures 1988). Piscivorous birds such as the great cormorant (*Phalacrocorax carbo sinensis*) and darter (*Anhinga novaehollandiae*) act as the definitive hosts of this parasite (Rusconi et al. 2022). *Eustrongylides* infections have been linked to avian mortality, with severe peritonitis causing deaths in blue herons (*Ardea herodias*) (Ziegler et al. 2000) and in egrets (Ciconiiformes) (Spalding and Forrester 1993), as well as documented pathogenicity in fish (Mihalca et al. 2007) and reptiles (Shamsi et al. 2023). As a cosmopolitan parasite (Mihalca et al. 2007), *Eustrongylides* can also infect humans through the accidental ingestion of L4 larvae, primarily from raw or undercooked fish, leading to damage of internal organs (Williams et al. 2022).

In the present study, larvae of *Eustrongylides* sp., isolated from the paratenic host *C. punctate*, were coiled, and the cephalic extremity appeared more reddish than the rest of the body, likely due to its attachment site, and was visible to the naked eye due to the thin cuticle layer of the larvae. These findings are consistent with those reported by Gupta (2019) and Noor et al. (2025). In anterior extremity of the parasites, two circles of papillae, inner and outer, were observed. The rounded posterior extremity with terminal anus and genital primordial, along with the morphometrical features permitted to designate the larvae to the genus *Eustrongylides*. Moreover, the infection site, shape and measurements of these larvae are similar to those found in previous reports by Guagliardo et al. (2019) for the same host and environment.

The morphological identification of different species of *Eustrongylides*, including *E. tubifex, E. ignotus* and *E. excisus*, was based on variations in adult labial papillae and the caudal extremity of males (Fusco et al. 2023). This approach underscores the difficulties in identifying species of *Eustrongylides* larvae isolated from fish. Larval stages, particularly L4, do not exhibit distinct morphological features that are seen in adults, and the larvae of different species are often morphologically indistinguishable (Mazzone et al. 2019; Xiong et al. 2009).

The combined molecular and morphological analyses supported the identification of the larvae at the genus level as *Eustrongylides* sp., thereby providing sequence data for this species. In the sequence identity matrix, the studied sequences exhibited high similarity (99.7%–99.8%) with the reference *Eustrongylides* sp. sequences (GQ215533.1 and GQ215560.1), suggesting a closer genetic relationship. The observed range of similarity (%) among these isolates indicates that they likely belong to the same taxon, aligning with the findings of Ozturk and Ozturk (2023). Comparisons of *16S rRNA* gene sequences from proposed and validated species have revealed identities exceeding the commonly cited 98.7% threshold used to define species boundaries (Caudill and Brayton 2022), highlighting inconsistencies in applying fixed similarity criteria. Although such thresholds are frequently referenced in taxonomic studies, they do not represent universally applicable standards. The approach based on sequence divergence remains inherently subjective and serves primarily as a heuristic reference rather than a definitive criterion for species delimitation. Reported variation thresholds differ among studies; for example, a 3% pairwise distance has been recommended by Schloss and Handelsman (2006), whereas Acinas et al. (2004) proposed a 1% cutoff. These discrepancies underscore the difficulty of establishing a single, universally robust sequence similarity threshold for species delineation. However, substitution rates and genetic variation can differ across lineages and species, making it difficult to apply a standardized cut‐off for taxonomic classification (Loren et al. 2018). Although sequence identity provides valuable insights into genetic relationships, species delimitation should be supported by multiple lines of evidence, including morphological, ecological and additional molecular data, to ensure robust taxonomic classification.

In this study, three distinct haplotypes were identified among 44 *Eustrongylides* spp. isolated from *C. punctata*, with an overall haplotype diversity of 0.0899 and only two SNPs detected. Although the presence of multiple haplotypes suggests some sequence variation, the extremely low nucleotide diversity indicates limited genetic differentiation. Therefore, any inference about geographic structuring should be considered preliminary. Similar patterns of low genetic variation have been reported in related studies (Ozturk and Ozturk 2023). Palm et al. (2008) identified three distinct haplotypes of anisakid nematodes in Brazil, differing by three to seven base pairs in the *ITS* region. According to Kellermanns et al. (2007), extensive migration of both final and intermediate hosts contributes to high gene flow. *ITS*2 gene has been widely used for nematode molecular identification (Dold and Holland 2014); reliance on a single nuclear marker may limit the resolution of species‐level delimitation (Dietz et al. 2023), particularly for larval stages. Future studies incorporating mitochondrial markers such as *cox1* or *nad1* would provide greater taxonomic resolution and confirm species boundaries within *Eustrongylides* populations in Bangladesh. Furthermore, more intense studies involving other fish host from different localities are needed to generalize these findings and to determine whether the observed differences in the *ITS1* region represent normal intraspecific variation or indicate a potential characteristic of *Eustrongylides* sp. in Bangladesh.

The phylogenetic analysis provides a confirmation for the molecular identification of the recovered nematodes as *Eustrongylides* sp. The studied isolates clustered with previously reported *Eustrongylides* sequences from Asia, particularly those from China and India, indicating a close genetic relationship among regional populations. Such genetic similarity among geographically related isolates may reflect a shared evolutionary origin and historical connectivity of parasite populations (Queiroz 2007). In contrast, sequences belonging to *E. ignotus* formed a relatively distinct lineage in the phylogenetic tree, suggesting evolutionary divergence among species within the genus. Genetic divergence among lineages may arise through processes such as geographic isolation, adaptation to different ecological conditions and genetic drift over evolutionary time (Felsenstein 2004). Phylogenetic trees provide a useful framework for visualizing these shared evolutionary histories and relationships among homologous sequences (Balaban et al. 2019). Environmental and geographic factors can also influence the genetic structure of parasite populations by affecting host movement and dispersal pathways, potentially limiting gene flow and promoting differentiation among populations (Clements et al. 2006; Rousset 1997; Bohonak 2002). Therefore, the present findings provide additional information on the diversity and evolutionary relationships of *Eustrongylides* spp. infecting freshwater fishes. However, further multi‐marker molecular studies are required to better understand the geographical distribution, epidemiology and taxonomic resolution of this parasite (Abe 2011).

## 5 Conclusion

The present study provides molecular and phylogenetic insights into *Eustrongylides* spp. isolated from *C. punctata* in Bangladesh, expanding upon previous reports by incorporating haplotype analysis in two different locations and broader phylogenetic comparisons. In the present study, three distinct haplotypes were identified, and two SNPs from 44 *Eustrongylides* spp. isolates were detected. The fourth‐stage larva of *Eustrongylides* spp. was identified by the morphological features and further validated by molecular tools. The data of genetic analysis based on partial sequence of *ITS2* gene could provide useful leads for further studies on *Eustrongylides* spp., including assessments of genetic diversity among different geographical locations. Hence, further comprehensive exploration involving a larger number of samples and employing diverse molecular markers is essential to enhance our understanding of the population genetic structure of this parasite. The results of this study may contribute to future efforts aimed at understanding the genetic characteristics of *Eustrongylides* species regionally and globally and could inform the development of prevention and control strategies in fisheries and aquaculture.

## Author Contributions

**Salman Shahriar Nibir**: formal analysis, software, investigation, writing – original draft, methodology. Anita Rani Dey: methodology, data curation, formal analysis, supervision, writing –review and editing, funding acquisition. **Tanvir Rahman**: conceptualization, validation, supervision, writing – review and editing. **Julfat Tasnim Suchona**: software, validation, investigation, data curation. **Siyam Hossain**: methodology, validation, formal analysis, writing – original draft. **Savayan Sadad Bushra**: software, data curation, investigation, formal analysis, writing – original draft.**Jubaida Parveen Juthy**: software, validation, investigation, formal analysis. **Sandhya Paul**: methodology, validation, investigation, writing – original draft. **Jarin Tasnim Tanwi**: methodology, software, formal analysis, writing – original draft. **Tamanna Tabassum**: methodology, software, validation, writing – original draft. **Md. Ali Reza Faruk**: software, formal analysis, investigation, writing – review and editing.

## Funding

This study was funded by Bangladesh Agricultural University Research System (BAURES), under grant number: 2021/113/BAU.

## Ethics Statement

Present study was conducted in accordance with the ethical standard of Research Committee of Bangladesh Agricultural Research System (BAURES), Bangladesh Agricultural University (BAU), Mymensingh 2202, Bangladesh. The approval reference number is BAURES/ESRC/53/FISH/2024, dated 31 December 2024.

## Conflicts of Interest

The authors declare no conflicts of interest.

## Supporting information




**Supporting File 1**: vms370965‐sup‐0001‐TableS1.docx.

## Data Availability

Data supporting this study are included within the article, and the raw data that support the findings of this study are available upon request from the corresponding author.
