# Supplementary material for: Molecular Characterization of Eustrongylides sp. Infecting Channa punctata in Bangladesh
Source: Vet Med Sci. 2026 Apr 29;12(3):e70965. doi: 10.1002/vms3.70965 (PMC13127553; doi:10.1002/vms3.70965)
Supplement: Supplementary file 1 — Supporting File 1: vms370965‐sup‐0001‐TableS1.docx. [file VMS3-12-e70965-s001.docx]

Table S1: List of sequences used in the phylogenetic analysis, including parasite names, host species, references, and geographical locations

| **GenBank Accession Number** | **Parasite names** | **Host** | **Geographic Location** | **Reference** |
| --- | --- | --- | --- | --- |
| GQ215577.1 | *Eustrongylides sp.* | *Hemibarbus maculatus* | China | Xiong et al. (2013) |
| GQ215535.1 | *Eustrongylides sp* | *Silurus asotus* | China | Xiong et al. (2013) |
| PP256050.1 | *Eustrongylides sp.* | *Alligator sinensis* | China | Shuai et al. (2025) |
| GQ215542.1 | *Eustrongylides sp.* | *Opsariichthys bidens* | China | Xiong et al. (2013) |
| GQ215543.1 | *Eustrongylides sp.* | *Silurus asotus* | China | Xiong et al. (2013) |
| MK545540.1 | *Eustrongylides excisus* | *Phalacrocorax carbo* | Italy | Unpublished |
| MK545536.1 | *Eustrongylides excisus* | *Phalacrocorax carbo* | Italy | Mazzone et al. (2019) |
| OP480439.1 | *Eustrongylides excisus* | *Sander lucioperca* | Turkey | Öztürk and Öztürk (2023) |
| MK545539.1 | *Eustrongylides excisus* | *Phalacrocorax carbo* | Italy | Unpublished |
| MK545492.1 | *Eustrongylides excisus* | *Perca fluviatilis* | Italy | Mazzone et al. [23] |
| OP480438.1 | *Eustrongylides excisus* | *Sander lucioperca* | Turkey | Öztürk and Öztürk (2023) |
| MK545538.1 | *Eustrongylides excisus* | *Phalacrocorax carbo* | Italy | Unpublished |
| MK545537.1 | *Eustrongylides excisus* | *Phalacrocorax carbo* | Italy | Unpublished |
| KU963206.1 | *Eustrongylides sp.* | *Esox lucius* | Iran | Unpublished |
| MK545499.1 | *Eustrongylides excisus* | *Perca fluviatilis* | Italy | Unpublished |
| MK545528.1 | *Eustrongylides excisus* | *Phalacrocorax carbo* | Italy | Unpublished |
| MK545532.1 | *Eustrongylides excisus* | *Phalacrocorax carbo* | Italy | Unpublished |
| MK545534.1 | *Eustrongylides excisus* | *Phalacrocorax carbo* | Italy | Unpublished |
| MK007967.1 | *Eustrongylides excisus* | *Sander lucioperca* | Turkey | Pekmezci and Bolukbas (2021) |
| MK545434.1 | *Eustrongylides excisus* | *Phalacrocorax carbo* | Italy | Unpublished |
| MK545531.1 | *Eustrongylides excisus* | *Phalacrocorax carbo* | Italy | Unpublished |
| MK545504.1 | *Eustrongylides excisus* | *Phalacrocorax carbo* | Italy | Unpublished |
| OK380960.1 | *Eustrongylides excisus* | *Perca fluviatilis* | Italy | Unpublished |
| PP333225.1 | *Eustrongylides excisus* | *Gambusia holbrooki* | Turkey | Eren et al. (2025) |
| GQ215576.1 | *Eustrongylides sp.* | *Hemibarbus maculatus* | China | Xiong et al. (2013) |
| GQ215529.1 | *Eustrongylides sp.* | *Channa argus* | China | Xiong et al. (2013) |
| LC708132.1 | *Eustrongylides sp.* | *Rhinogobius sp.* | Japan | Unpublished |
| GQ215573.1 | *Eustrongylides sp.* | *Coilia nasus* | China | Xiong et al. (2013) |
| GQ215528.1 | *Eustrongylides sp.* | *Channa argus* | China | Xiong et al. (2013) |
| GQ215550.1 | *Eustrongylides sp.* | *Silurus asotus* | China | Xiong et al. (2013) |
| GQ215536.1 | *Eustrongylides sp.* | *Silurus asotus* | China | Xiong et al. (2013) |
| GQ215536.1 | *Eustrongylides sp.* | *Silurus asotus* | China | Xiong et al. (2013) |
| GQ215532.1 | *Eustrongylides sp.* | *Monopterus albus* | China | Xiong et al. (2013) |
| GQ215560.1 | *Eustrongylides sp.* | *Monopterus albus* | China | Xiong et al. (2013) |
| GQ215569.1 | *Eustrongylides sp.* | *Channa argus* | China | Xiong et al. (2013) |
| KJ458967.1 | *Eustrongylides sp.* | *Channa punctatus* | India | Kundu and Mandal (2022) |
| GQ215570.1 | *Eustrongylides sp.* | *Channa argus* | China | Xiong et al. (2013) |
| GQ215503.1 | *Eustrongylides sp.* | *Monopterus albus* | China | Xiong et al. (2013) |
| GQ215508.1 | *Eustrongylides sp.* | *Monopterus albus* | China | Xiong et al. (2013) |
| GQ215552.1 | *Eustrongylides sp.* | *Monopterus albus* | China | Xiong et al. (2013) |
| GQ215559.1 | *Eustrongylides sp.* | *Monopterus albus* | China | Xiong et al. (2013) |
| GQ215531.1 | *Eustrongylides sp.* | *Monopterus albus* | China | Xiong et al. (2013) |
| GQ215530.1 | *Eustrongylides sp.* | *Monopterus albus* | China | Xiong et al. (2013) |
| GQ215557.1 | *Eustrongylides sp.* | *Monopterus albus* | China | Xiong et al. (2013) |
| GQ215502.1 | *Eustrongylides sp.* | *Monopterus albus* | China | Xiong et al. (2013) |
| OP830362.1 | *Eustrongylides excisus* | *Maccullochella peelii* | Australia | Shamsi et al. (2023) |
| OP830363.1 | *Eustrongylides excisus* | *Maccullochella peelii* | Australia | Shamsi et al. (2023) |
| OP830361.1 | *Eustrongylides excisus* | *Maccullochella peelii* | Australia | Shamsi et al. (2023) |
| EU394732.1 | *Eustrongylides ignotus* | *Gambusia affinis* | USA | Unpublished |
| KT013207.1 | *Eustrongylides ignotus* | *Xiphophorus variatus* | Taiwan | Unpublished |
| MK340916.1 | *Eustrongylides ignotus* | *Culter alburnus* | China | Shamsi et al. (2023) |
| KF748494.1 | *Xiphinema americanum* | *Vaccinium sp.* | USA | Zasada et al. (2014) |
